# Expression patterns, mutation detection and RNA interference of *Rhopalosiphum padi* voltage-gated sodium channel genes

**DOI:** 10.1038/srep30166

**Published:** 2016-07-21

**Authors:** Yayun Zuo, Xiong Peng, Kang Wang, Fangfei Lin, Yuting Li, Maohua Chen

**Affiliations:** 1 College of Plant Protection, Key Laboratory of Crop Pest Integrated Pest Management on the Loess Plateau of Ministry of Agriculture, Northwest A&F University, Yangling, Shaanxi, 712100, P.R. China

## Abstract

The voltage-gated sodium channel (VGSC) is the target of sodium-channel-blocking insecticides. Traditionally, animals were thought to have only one VGSC gene comprising a α-subunit with four homologous domains (DI–DIV). The present study showed that *Rhopalosiphum padi*, an economically important crop pest, owned a unique heterodimeric VGSC (H1 and H2 subunits) encoded by two genes (*Rpvgsc1* and *Rpvgsc2*), which is unusual in insects and other animals. The open reading frame (ORF) of *Rpvgsc1* consisted 1150 amino acids, and the ORF of *Rpvgsc2* had 957 amino acids. Rpvgsc1 showed 64.1% amino acid identity to DI–DII of *Drosophila melanogaster* VGSC and Rpvgsc2 showed 64.0% amino acid identity to DIII–DIV of *D. melanogaster* VGSC. A M918L mutation previously reported in pyrethroids-resistant strains of other insects was found in the IIS4-S6 region of *R. padi* field sample. The two *R. padi* VGSC genes were expressed at all developmental stages and showed similar expression patterns after treatment with beta-cypermethrin. Knockdown of *Rpvgsc1* or *Rpvgsc2* caused significant reduction in mortality rate of *R. padi* after exposure to beta-cypermethrin. These findings suggest that the two *R. padi* VGSC genes are both functional genes.

Pyrethroid insecticides are commonly used in pest insects control, and voltage-gated sodium channel (VGSC), as the primary target of pyrethroids, have been studied in many insect species^1^,^2^. Pyrethroids alter the gating kinetics of the channel by slowing both activation and inactivation, resulting in repetitive discharges that lead to overstimulation of the nerve and eventually to paralysis and death^1^,^2^. For example, beta-cypermethrin prolongs the opening of sodium channels, resulting in repetitive nerve firing and membrane depolarization in insects^3^.

Traditionally, animals are thought to have only one sodium channel gene, with alternative splicing of exons imparting functional variability^1^,^4^. For mammals, insects and other animals, the α-subunit sodium channel is the major structural subunit of the inset sodium channel and comprises four homologous domains (I–IV), each of which includes six transmembrane (TM) segments (S1–S6) expressed in different tissues and/or developmental stages^1^,^4^,^5^. The selectivity filter and pore of VGSC are formed by transmembrane segments S5 and S6, together with the membrane-reentrant segments that are part of the loop connecting S5 and S6 of each domain^1^,^5^. The amino acids DEKA (Asp, Glt, Lys, and Ala) in the loop connecting the S5 and S6 of domains I, II, III, and IV, respectively, are thought to mediate the Na^+^ selectivity of the channel^1^,^6^. However, a very recent report found that the VGSC genes in two aphid species, *Acyrthosiphon pisum* and *Myzus persicae*, were encoded by two separate genes (one encoding DI-II, the other DIII-IV), and the selectivity filter was found to be DENS (Asp, Glu, Asn, and Ser) (domains I–IV) rather than the standard sodium-selective DEKA filter^7^.

Many pests have developed resistance to pyrethroid insecticides due to their intensive use. Pyrethroid resistance in insects is conferred by mutations in the voltage-gated sodium channel (VGSC) gene and increased activity of detoxification enzymes, such as cytochrome P450s^8^. More than 50 unique resistance-associated mutations or combinations of mutations in VGSC genes have been detected in several species, and the majority of functionally confirmed mutations are found in the domain IIS4–S6 region^1^,^4^, in which five mutation sites (M918, L925, T929, F932, L1014) associated with pyrethroid resistance have been identified in several species, including *M. persicae*^9^, *Aphis gossypii*^10^,^11^, and *Sitobion avenae*^12^.

*Rhopalosiphum padi* (Linnaeus) (Hemiptera: Aphididae) is one of the key pests of wheat crops, causing significant damage by direct feeding and by the transmission of plant viruses^13^. This aphid is distributed in wheat-growing areas worldwide. In China, damage by *R. padi* is increasing annually in wheat-growing regions^14^,^15^. Resistance of *R. padi* to various insecticides, including pyrethroids, has been reported in China^16^,^17^.

Despite the economic importance of *R. padi* and development of insecticide resistance of the pest, the molecular mechanisms underlying its pyrethroid resistance are still unknown. Our objective was to clone and analyze the structural features of the *R. padi* VGSC gene (*Rpvgsc*), and assess the expression of *Rpvgsc* at different developmental stages of the aphid. We analyzed the transcriptional characteristics of the VGSC genes in *R. padi* after exposure to beta-cypermethrin and detected mutation sites in the IIS4–IIS6 region of *Rpvgsc* from field populations. We hypothesized that *R. padi* harbors two *Rpvgsc* genes, both of which are neurotargets. RNA interference (RNAi) was used to test the function of VGSC genes in *R. padi*.

## Results

### cDNA cloning and characterization of *Rpvgsc* genes

**Figure 1:**
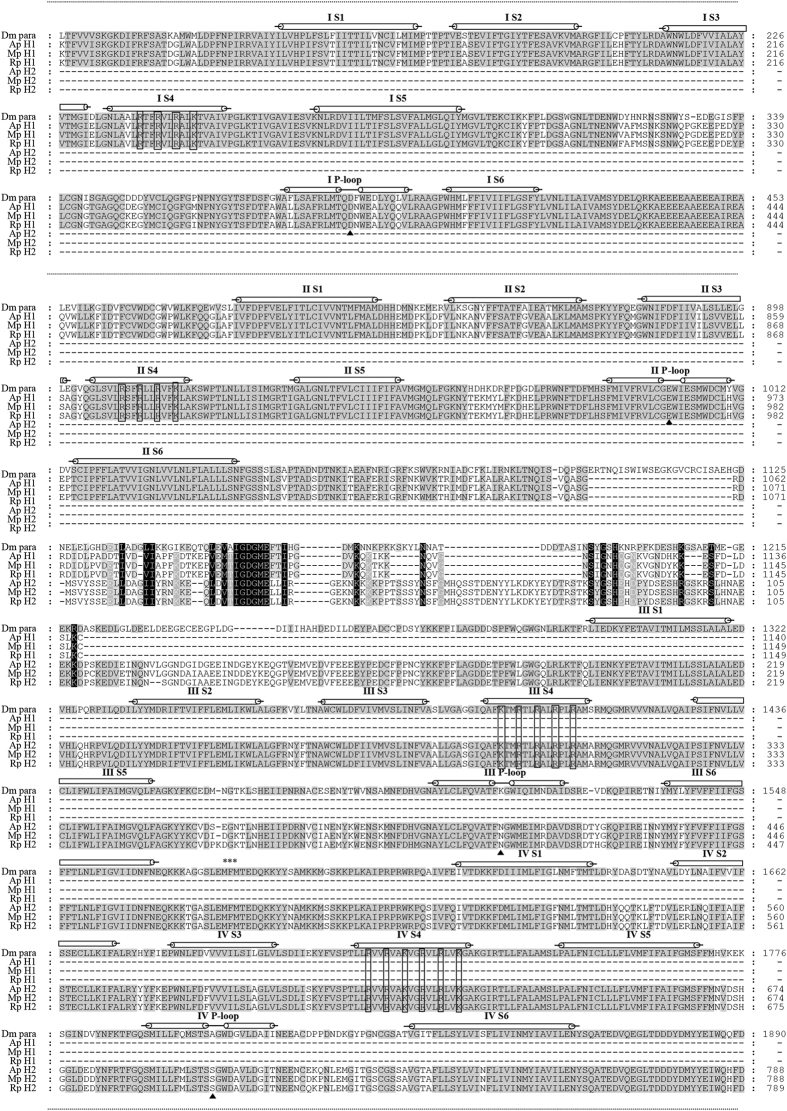
Alignment of deduced amino acid sequences of the *R. padi* voltage-gate sodium channel gene H1 and H2 subunits with sodium channels of *D. melanogaster* and with the H1 and H2 subunits of the *A. pisum* and *M. persicae* sodium channels. Transmembrane helices from different domains are indicated by bars above the sequences. Identical amino acids are highlighted in gray. The apparent evolutionary sequence duplication in the DII-DIII intracellular loop to generate the novel 5′ end for H2 is highlighted in black. The MFM motif is indicated by an asterisk (*). Basic amino acid residues in helix 4 are indicated by a black box. Triangles mark the positions of the sodium selectivity filter motif (DENS). The amino acid sequences far from the six transmembrane segments (S1–S6) are indicated by the suspension point lines. (−), deletion; Dm para, VGSC amino sequences of *Drosophila melanogaster* (GenBank accession No.: AAB59195.1); Ap H1, amino sequences of *Acyrthosiphon pisum* VGSC H1 subunit (XP_008183365.1); Mp H1, amino sequences of *Myzus persicae* VGSC H1 subunit (CBI71141.1); Rp H1, amino sequences *of Rhopalosiphum padi* VGSC H1 subunit (KJ872633); Ap H2, amino sequences of *Acyrthosiphon pisum* VGSC H2 subunit (XP_001949648.2); Mp H2, amino sequences of *Myzus persicae* VGSC H2 subunit (CBI71142.1); Rp H2, amino sequences *of Rhopalosiphum padi* VGSC H2 subunit (KP966088).

We obtained two *Rpvgsc* genes, *Rpvgsc1 and Rpvgsc2*, which encoded the H1 and H2 subunits, respectively. The complete open reading frame (ORF) of the *Rpvgsc1* was 3450 nucleotides long, encoding 1150 amino acids (~13.03 kDa) (GenBank accession no.: KJ872633). The *Rpvgsc2* ORF was 2874 nucleotides long, encoding 957 amino acids (~11.01 kDa) (GenBank accession no.: KP966088). The *Rpvgsc1* and *Rpvgsc2* ORFs suggested that the *R. padi* sodium channel was encoded by two unique 2 × 6 TM heteromers. Rpvgsc1 had 64.1% identity to the amino acid sequence of the DI–DII domain of the *Drosophila melanogaster* para voltage-gate sodium channel gene, whereas the Rpvgsc2 had 64.0% identity to the amino acid sequence of the DIII–DIV domain of the *D. melanogaster* para voltage-gate sodium channel gene (Fig. 1). The predicted *R. padi* Rpvgsc1 and Rpvgsc2 possessed the expected features of a voltage-dependent sodium channel (Fig. 1). First, the S4 region of each domain in *R. padi* Rpvgsc1 and Rpvgsc2 had four to seven basic amino acid residues, arginine (R) or lysine (K), separated by two neutral amino acid residues. These sequence features are characteristics of voltage sensors, which move outward in response to membrane depolarization and initiate the opening of the channel^18^. Second, a conserved insect MFM motif, which is conserved in invertebrate VGSC and corresponds to the IMF motif in the mammalian sodium channel and is critical for rapid inactivation of the sodium channel^18^, was found in the linker between domains III and IV in *R. padi Rpvgsc2*. Third, a channel selectivity filter motif (DENS) was found in *R. padi* at the position of the sodium selective DEKA filter in other eukaryotes^19^. The sodium selective filter was formed by one amino acid from each of the four P-loops and is critical for sodium selectivity^19^.

**Figure 2:**
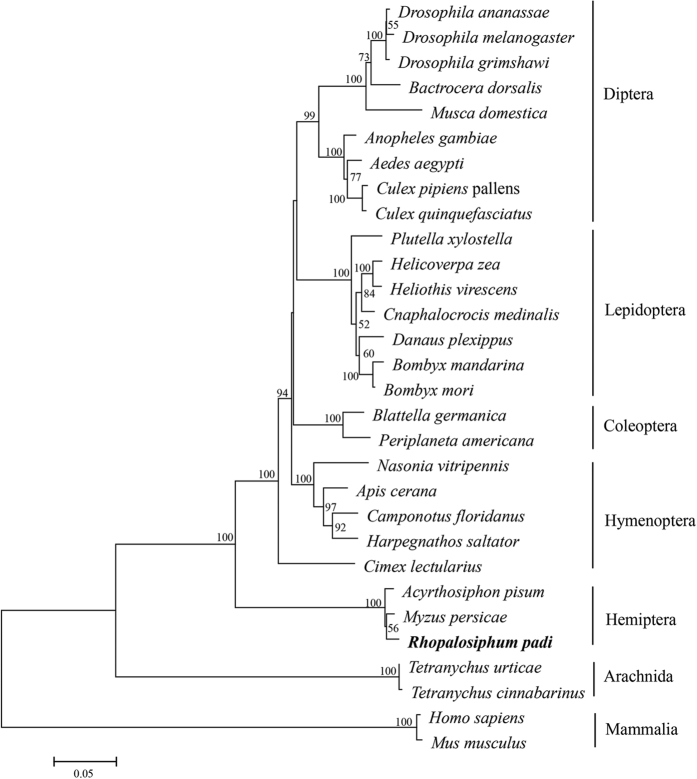
Phylogenetic analysis of *Rpvgsc* with other VGSC genes from arthropods and mammalians. Values at the nodes indicate bootstrap support values based on 1000 replicates. Branches with bootstrap values above 50% were collapsed. GenBank accession numbers or Gene IDs are as follows: *Drosophila ananassae* (XP_001966146.1), *Drosophila melanogaster* (AAB59195.1), *Drosophila grimshawi* (XP_001992511.1), *Bactrocera dorsalis* (AEX08661.1), *Musca domestica* (CAA65448.1), *Anopheles gambiae* (CAM12801.1), *Aedes aegypti* (ACB37023.1), *Culex pipiens pallens* (BAI77918.1), *Culex quinquefasciatus* (CAM31893.1), *Plutella xylostella* (BAF37096.2), *Helicoverpa zea* (ADF80418.1), *Heliothis virescens* (AAC26517.1), *Cnaphalocrocis medinalis* (AGH70334.1), *Danaus plexippus* (EHJ74501.1), *Bombyx mandarina* (ACD80425.1), *Bombyx mori* (NP_001136084.1), *Blattella germanica* (AAC47484.1), *Periplaneta americana* (ACX44801.1), *Nasonia vitripennis* (XP_008204157.1), *Apis cerana* (AEY56607.1), *Camponotus floridanus* (EFN61422.1), *Harpegnathos saltator* (EFN86793.1), *Cimex lectularius* (ACI43362.1), *Myzus persicae* (CBI71141.1 and CBI71142.1), *Acyrthosiphon pisum* (XP_008183365.1 and XP_001949648.2), *Rhopalosiphum padi* (KJ872633 and KP966088), *Tetranychus urticae* (AFU35097.1), *Tetranychus cinnabarinus* (AFR68409.1), *Homo sapiens* (NP_066287.2), *Mus musculus* (NP_001092768.1). The variable regions between the H1 and H2 subunits of the three aphid species—*R. padi*, *A. pisum*, and *M. persicae*—were excluded, and the two subunits were combined for better alignment. Bootstrapping of the ML analysis was conducted using 1000 replicates.

The phylogenetic tree exhibited branches formed by the respective VGSCs from mammalian, insecta and arachnida. VGSC from *R. padi* and two other aphid species formed a subbranch, which was differentiated from other insect VGSCs (Fig. 2). *Rpvgsc* genes along with other those of insect species shared a common ancestor gene, suggesting that *Rpvgsc* genes were para-orthologous (Fig. 2).

### Mutations in the IIS4–IIS6 region of the *R. padi Rpvgsc* gene

**Figure 3:**
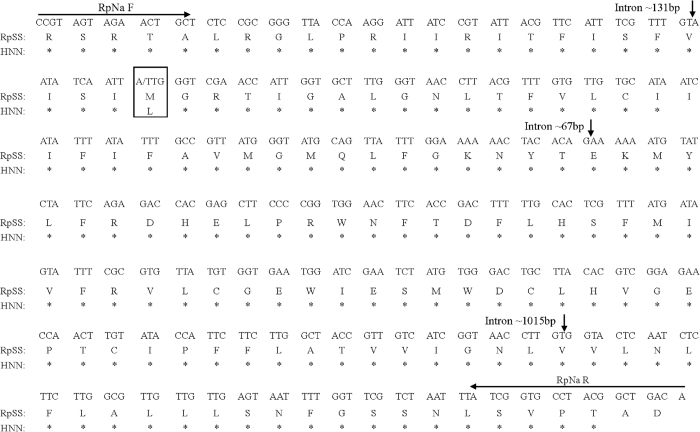
Nucleotide and amino acid sequences of the IIS4–IIS6 region from the susceptible *R. padi* clone (RpSS) and a field population (HNN). The single codon change ATG to TTG causing the super-kdr substitution M918L is boxed. The positions of the PCR and sequencing primers are indicated by arrows. The positions of the three introns (intron 1, ~131 bp; intron 2, ~67 bp; and intron 3, ~1010 bp) are indicated by vertical arrows.

**Figure 4:**
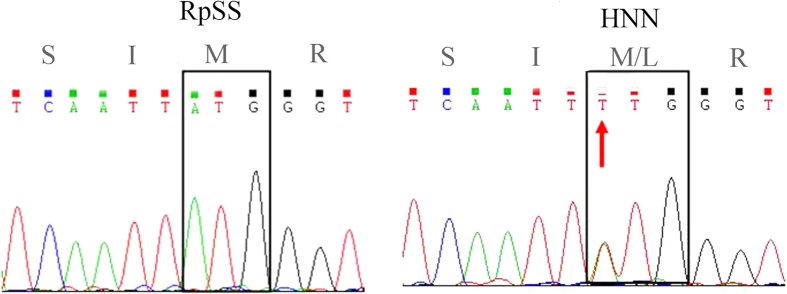
Sequencing chromatogram for the *R. padi* sample from the susceptible clone (RpSS) and for the individual from a field population (HNN) harboring the M918L mutation. Two peaks (representing ATG and TTG) overlap, indicating a heterozygous mutation.

The IIS4-6 region (~1640 bp) of the *R. padi* VGSC gene was amplified by RT-PCR using the RpNa primers (Table 1). Three introns (intron 1, ~131 bp; intron 2, ~67 bp; and intron 3, ~1010 bp) were present in this region. No *R. padi* field samples had mutation in the extron of the IIS4-6 region except that only one individual from the HNN population showed a single base difference, which caused a super-*kdr* mutation (M918L) (Fig. 3). This base change (ATG to TTG) appeared as a mixed peak in the sequence chromatograms (Fig. 4), indicating a heterozygous allele. No other mutation sites (M918, L925, T929, and F932) previously reported in other insect species were detected in the samples.

### Expression of *Rpvgsc* genes at different developmental stages of *R. padi*

**Figure 5:**
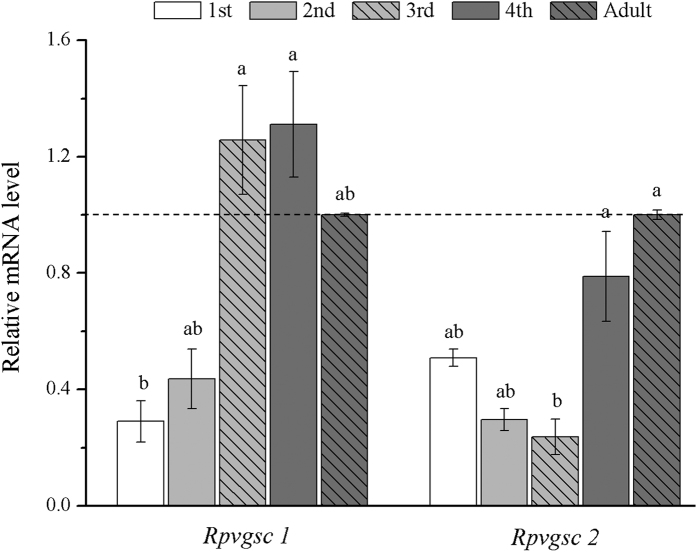
Relative *Rpvgsc1* and *Rpvgsc2* expression levels at different developmental stages of *R. padi*. Expression levels were normalized using *β-actin* as the standard. The normalized value was applied to relative expression analysis. The results are means ± SE. Different letters on the error bars indicate significant differences between the developmental stages according to ANOVA followed by Tukey’s HSD multiple comparison (*p* < 0.05).

qRT-PCR analysis showed that *Rpvgsc1* and *Rpvgsc2* were expressed at all developmental stages of *R. padi*. The highest expression level of *Rpvgsc1* was found in the 3rd instar and was 1.14-fold of that in the adult aphid (Fig. 5). The lowest expression level was found in the 1st instar and was significantly lower than those at all other developmental stages (*p* < 0.05) (Fig. 5). There were no significant differences among *Rpvgsc1* gene expression levels in the 2nd, 3rd, 4th, and adult. The highest expression level of *Rpvgsc2* was found in 4th instar *R. padi*, and the lowest expression level was found in the 3rd instar. There was no significant difference in the *Rpvgsc2* expression level among the 1st, 2nd, 4th, and adult (Fig. 5). In addition, the *Rpvgsc1* and *Rpvgsc2* expression levels differed significantly in the 3rd instar.

### Expression of *Rpvgsc* genes upon exposure to beta-cypermethrin

**Figure 6:**
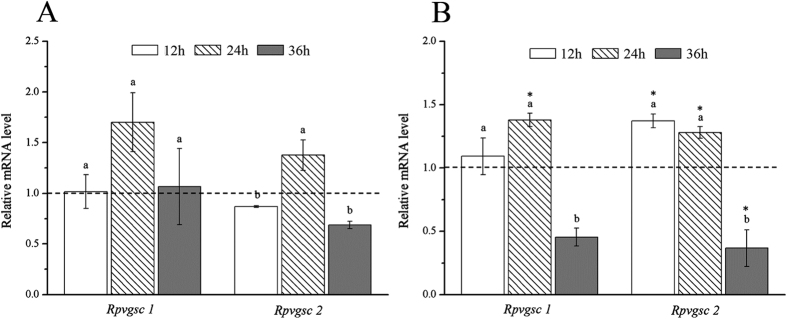
Expression of *Rpvgsc1* and *Rpvgsc2* following exposure to beta-cypermethrin. (**A**) Exposure to 0.3987 mg/L beta-cypermethrin. (**B**) Exposure 0.9280 mg/L beta-cypermethrin. Expression levels were first normalized to that of the reference gene β*-actin*. Relative transcription level = transcription level under stress treatment/transcription level under control conditions. The resulting ratio was applied to relative expression analysis. The results are means ± SE. Different letters above bars indicate significant differences in *Rpvgsc1* and *Rpvgsc2* expression levels at the indicated hours post-exposure based on ANOVA followed by Tukey’s HSD multiple comparison (*p* < 0.05). Asterisks above bars indicate significant differences between the treatment and the corresponding control (*p* < 0.05).

Compared with the control, the *Rpvgsc1* and *Rpvgsc2* expression levels were influenced by exposure to LC_10_ and LC_30_ concentrations of beta-cypermethrin. When exposed to 0.3987 mg/L beta-cypermethrin, the transcription levels of *Rpvgsc1* and *Rpvgsc2* peaked at 24 h and were 1.70-fold and 1.38-fold, respectively (Fig. 6A). There was no significant difference in *Rpvgsc1* expression levels among the treatment time points. The transcription level of *Rpvgsc2* at 24 h was significantly higher than that at 12 h and 36 h (Fig. 6A). Upon exposure to 0.9280 mg/L beta-cypermethrin, the transcription levels of *Rpvgsc1* (at 24 h) and *Rpvgsc2* (at 12 h) peaked; both were around 1.37-fold of that for the control (Fig. 6B). The transcription levels of *Rpvgsc1* and *Rpvgsc2* at 36 h were significantly lower than those at 12 h and 24 h (Fig. 6B). Upon exposure to 0.3987 mg/L and 0.9280 mg/L beta-cypermethris, there was no significant difference in *Rpvgsc1* and *Rpvgsc2* expression throughout the treatment period (Fig. 6).

### RNAi of H1 and H2 subunit genes and the effect on *R. padi* susceptibility to beta-cypermethrin

**Figure 7:**
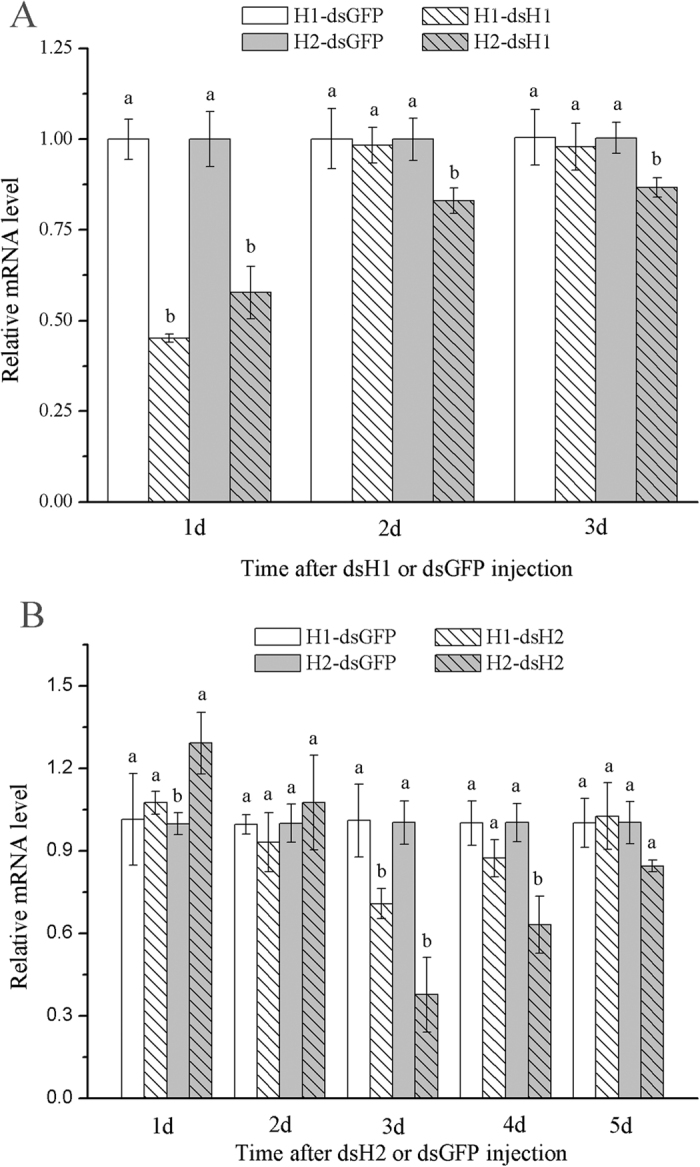
The expression levels of H1 and H2 subunit genes in *R. padi* after H1-dsRNA injection (**A**) and H2-dsRNA injection (**B**). The levels of H1 and H2 subunit genes transcription in aphids injected with H1- or H*2*-dsRNA were normalized relative to which in aphids injected with dsGFP. Data were presented as mean ± SE. Different letters above bars indicate significant differences based on ANOVA followed by Tukey’s HSD multiple comparison (*p* < 0.05).

The silencing efficiencies of the two dsRNAs on H1 and H2 genes were examined using real-time quantitative PCR. Injection of H1-dsRNA and H2-dsRNA was effective in silencing the respective transcription of H1 and H2 subunit genes. The H1 subunit gene mRNA levels decreased dramatically (reduced by 54.8%) at day 1 after injection of H1-dsRNA compared with the GFP- dsRNA injection, and expression of H2 subunit gene was reduced by 42.3%, indicating significant cross-suppressions in the transcript levels between H1 and H2 subunit genes. The RNAi effect of H1-dsRNA was greatly decreased at day 2 and day 3, and H1 subunit genes mRNA levels were similar to that in the control (Fig. 7A). Injection of H2-dsRNA also induced reduction in the transcript levels of both H1 and H2 subunit genes in *R. padi* (Fig. 7B). The transcript levels of H2 subunit gene were significantly reduced at day 3 after H2-dsRNA injection, with significant cross-suppressions in the transcript levels between H1 and H2 subunit genes at day 3, and the effect was still strongly at day 4.

**Figure 8:**
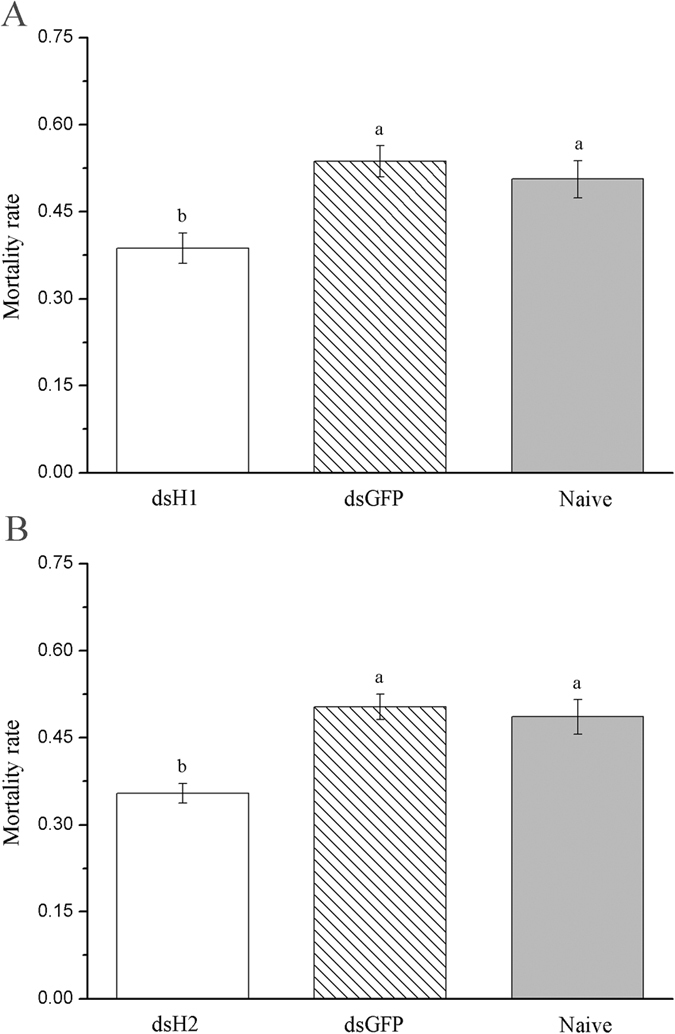
Mortality of *R. padi* apterous adults treated with LC_50_ concentrations of beta-cypermethrin after injection with H1-dsRNA (**A**) and H2-dsRNA (**B**). Data were presented as mean ± SE. Different letters above bars indicate significant differences based on ANOVA followed by Tukey’s HSD multiple comparison (*p* < 0.05).

RNAi of H1 and H2 subunit genes significantly decreased the susceptibility of *R. padi* to beta-cypermethrin (Fig. 8). After knockdown of H1 subunit gene, the mortality rate (38.7%) of the survivals was significantly lower than that of dsGFP injection and that of control (naive insect without any injection) after exposure to LC_50_ concentration of beta-cypermethrin (*p* < 0.05) (Fig. 8A). Similar result was observed after knockdown of H2 subunit gene (Fig. 8B).

## Discussion

Sodium channels are targeted by pyrethroids^20^. Due to the intensive use of pyrethroids, pyrethroid resistance, which involves reduced target-site sensitivity, has developed in many pest populations in the past several decades^1^. In this study, we cloned and characterized sodium channel genes from the important wheat pest *R. padi* and found that the *R. padi* para sodium channel was encoded by two separate genes. qRT-PCR analysis showed that the expression of the two genes varied at different *R. padi* developmental stages, and exposure to beta-cypermethrin affected the expression of both genes. The transcript levels of the two separate genes could be significantly suppressed by injection of their respective dsRNA. Knockdown each of the two genes significantly reduced the mortality rate of *R. padi* after exposure to LC_50_ concentrations of beta-cypermethrin.

*Rpvgsc* is an unique heterodimeric voltage-gated sodium channel encoded by two separate genes, each of which comprises two unique 2 × 6 TM heteromers and has a reasonable degree of identity with known para orthologs and the channel has the novel selectivity filter motif DENS rather than the DEKA of other eukaryotes. In a recent report, it was found that the VGSC of *M. persicae* was also encoded by, and assembled from two unique 2 × 6 TM heteromers^7^,^21^. In *A. pisum* genome, there were two-subunit sodium channel genes oriented in opposite directions on scaffold 318, separated by ~23 kb of non-coding sequence^7^. Therefore, the VGSC of aphids likely has a two-subunit channel.

The M918 mutation is consistently detected in the presence of the L1014 mutation in most insect species. We detected the VGSC M918L point mutation in a field population of *R. padi*, but did not find any mutation in L1014. The M918L mutation was previously reported to be responsible for the reduced sensitivity of VGSC to pyrethroids in resistant populations of two aphid species: *M. persicae*^9^ and *A. gossypii*^11^. M918V was detected in resistant populations of *B. tabaci*^22^ as a single mutation. The M918 or L1014 mutations associated with pyrethroid-resistance in the H1 subunit of *M. persicae*^9^, *A. gossypii*^10^,^11^, and *S. avenae*^12^ indicate that the H1 subunit of aphids is a neurotarget of pyrethroids. The low rate of mutation found in the field populations of *R. padi* may have been due to the fitness cost of mutations. Many researches had reported that a fitness cost associated with resistant individuals of insects was detected in field populations involving a high energetic cost or significant disadvantage^23^. As a consequence of the high fitness cost and rapid reproduction of the aphid, the rate of resistant individuals of *R. padi* with mutations could decline rapidly after a few generations in the absence of insecticide exposure^24^. In the last decade, neonicotinoid insecticides which targeted nicotinic acetylcholine receptors, were mainly used to control *R. padi* instead of pyrethroids in China, which can explain that M918 mutation was rare in the samples analyzed in current study.

Voltage-gated Na^+^ channels are considered to be members of a superfamily of channels that includes voltage-gated K^+^ channels, voltage-gated Ca^2+^ channels, and cyclic-nucleotide-gated channels^25^,^26^,^27^. The most widely accepted hypothesis is that the primordial Ca^2+^ channel (containing four homologous domains) evolved from K^+^ channels (consisting of tetramers of single-domain subunits) by two intragenic duplications, and then, for the reasons reviewed by Hille^28^, the Ca^2+^ channel gave rise to the Na^+^ channel by further divergence following gene duplication. Based on sequence similarities, Strong *et al*.^29^ suggested that the two-domain channel consisting of domains I/III and II/IV, each of which then duplicated to result in the first four-domain Ca^2+^ channel, emerged because of the original duplication event. Phylogenetic analysis suggested that the VGSC of aphids forms an independent branch, which is differentiated from other channel genes of insecta and arachnida species, but that it shared a common ancestor gene with the channel genes of other insecta species, suggesting that the aphid VGSC was para orthologous to VGSC of other insects and that aphid heterodimeric assembly had arisen by structural modification of an ancestral 4 × 6TM invertebrate Nav channel^7^. It is possible that gene fission resulted in this modification, which occurred in a duplication of part of the domain II–III linker region in the ancestral gene of aphids^7^.

**Figure 9:**
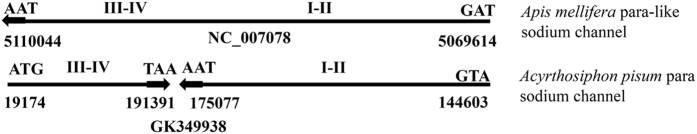
Genome positions of the *Apis A. mellifera para*-like sodium channel (upper panel) and *Acyrthosiphon pisum. para* sodium channel (lower panel) gene.

The *Apis mellifera* para-like sodium channel is encoded by one gene located on the LG9 chromosome (NC_007078: 5110044-5069614), and the pea aphid *A. pisum* para-like sodium channel is encoded by two genes located on scaffold318 [GL349938: 191764-191391 (III–IV) and 175077-144603 (I–II)], respectively. The two genes are transcribed in opposite directions, which suggests that the aphid para-like sodium channel is encoded by two genes. Modification of the aphid para-like sodium channel gene was caused by chromosome inversion (Fig. 9). *Rpvgsc1* and *Rpvgsc2* expression profiles were different in different stages. Knockdown one of the two genes could cause significant cross-suppressions of another one. On the other hand, the *Rpvgsc1* and *Rpvgsc2* expression levels were affected by treatment with two concentrations of beta-cypermethrin. Knockdown *Rpvgsc1* or *Rpvgsc2* could significantly reduce the susceptibility of *R. padi* to beta-cypermethrin, indicating the decrease of the primary (i.e. voltage-gated sodium channel) targets of pyrethroid reduce the effect of the chemical in overstimulation of the aphid nerve that eventually caused death. *Rpvgsc1* and *Rpvgsc2* transcription was up- and down-regulated at different treatment time points after exposure to beta-cypermethrin, which may maintain the ion equilibrium status of the body of *R. padi*. The aforementioned results suggested that *Rpvgsc1* and *Rpvgsc2* were both functional, and function together rather than individually. It is possible that expression of two genes was regulated by the different regulatory factors^7^. In different development stages of *R. padi*, the regulatory factors could regulate the corresponding gene differently, resulting in varied expression of two genes at a same stage. Further investigation is required to analyze the detailed co-expression patterns and the functions of *Rpvgsc1* and *Rpvgsc2* in *R. padi*, as well as to elucidate the adaptive evolution of voltage-gated sodium channel in *R. padi* and other aphid species.

## Materials and Methods

### Insects

A susceptible *R. padi* clone (RpSS) was reared in the laboratory for >3 years at 22 °C ± 1 °C with a 16 h light/8 h dark cycle and 80% relative humidity.

To identify mutations, apterous *R. padi* adults were collected from wheat (*Triticum aestivum* L.) in seven regions of various provinces of China (Table 2). One aphid was collected per plant, and the distance between the sampled plants was ≥30 m. The samples were preserved in absolute ethanol and transported to the laboratory for DNA extraction.

### *Rpvgsc* gene cloning

Total RNA was extracted from 15 apterous adult females from RpSS strain using 500 μL TRIzol reagent (Invitrogen, Carlsbad, CA) and treated with DNase I (Takara, Kyoto, Japan) to remove genomic DNA contamination. The purity and concentration were determined using a biophotometer (Eppendorf BioPhotometer Plus, Eppendorf, Germany). cDNA was synthesized from 2 μg of total RNA at 42 °C for 90 min with oligo dT-adaptor primers using an M-MLV reverse transcriptase cDNA Synthesis Kit according to the instructions of the manufacturer (Promega, Madison, WI, USA).

**Figure 10:**
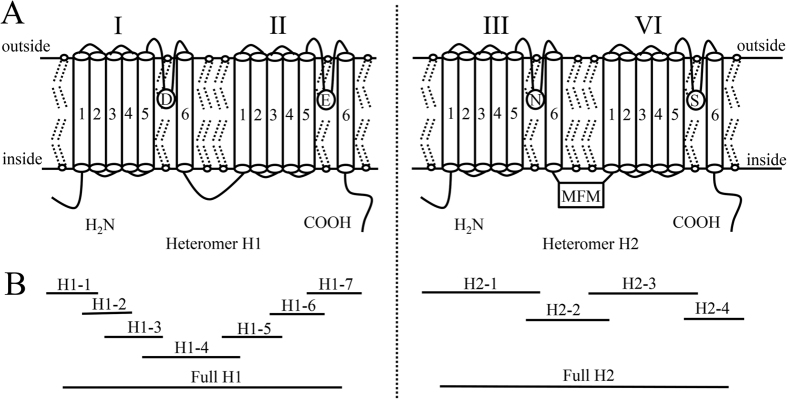
Schematic representation of the aphid sodium channel (**A**) and cloning strategies for the *R. padi* voltage-gate sodium channel gene (**B**). (**A**) The aphid sodium channel contains H1 and H2 subunits, each of which contains two homologous domains, which consist of six transmembrane regions (4 × 6 TM). The approximate position of the fast inactivation particle ‘MFM’ motif and ion (Na^+^) selectivity filter consists of one loop from each domain (located between S5 and S6 of the pore region), which collectively form a ‘DENS’ (DI-aspartate; DII-glutamate; DIII-asparagine; DIV-serine) amino acid sequence motif at the entry to the pore. (**B**) The locations and lengths of the cDNA fragments amplified in this study are shown.

*Rpvgsc* was amplified from cDNA. PCR was performed using gene-specific primers designed based on the nucleotide sequences of *vgsc* from *M. persicae* (accession nos. FN601405.1 and FN601406.1) and *A. pisum* (accession nos. XM_001949613.3 and XM_008185143.1) (Table 1). The amplification strategies and sequence-specific primers used for cloning *Rpvgsc* are shown in Fig. 10 and Table 1. We amplified 10 fragments (fragments H1-1 to H1-6 and H2-1 to H2-4) covering the open reading frames of two separate voltage-gated sodium channel genes. Then, two pairs of primers (Full H1 and Full H2; Table 1) were used to confirm the full length of the two genes, separately. PCR was performed in a volume of 25 μL using standard procedures with 2 μL of each primer (10 mmol L^−1^), 2.5 μL of 10× buffer (Mg^2+^ Plus), 4 μL of dNTPs (0.2 mmol L^−1^ each), 2 μL cDNA as the template, 0.25 μL of Takara LA-*Taq* DNA polymerase (LA Taq; Takara Bio, Dalian, China) (5 U/μL), and 12.25 μL of ddH_2_O, which resulted in more efficient amplification and higher fidelity than conventional *Taq* DNA polymerase, under PCR conditions of 94 °C for 4 min, followed by 30 cycles of 94 °C for 30 s, 52–62 °C for 30 s, and 72 °C for 1–4 min, and a final 10 min at 72 °C. The PCR products were purified using a Wizard PCR Preps kit (Promega, Madison, WI). The purified fragments were then cloned into the pGEM-Teasy Vector (Promega) and transformed into *Escherichia coli* JM109. Finally, five positive clones were randomly chosen for bidirectional sequencing on an Applied Biosystems 3730 automated sequencer (Applied Biosystems, Foster City, CA, USA) using the commercial services of Sangon Biotech (Shanghai, China).

### Detection of mutations in the IIS4–IIS6 region of *Rpvgsc*

Ten individuals from each of the seven regions were selected randomly as well as from the RpSS colne. Genomic DNA was extracted from single aphids using the DNeasy Tissue Kit following the manufacturer’s recommendations (Qiagen, Hilden, Germany). DNA was eluted using TE buffer and stored at −20 °C. The RpNa primer pair (Table 1) was used to amplify the IIS4-6 region of the *R. padi* sodium channel gene. PCR amplification, gel extraction, cloning, and plasmid purification were performed according to the aforementioned methods. Three positive clones from each sample were selected randomly for bidirectional sequencing on an Applied Biosystems 3730 automated sequencer (Applied Biosystems, Foster City, CA, USA) using the commercial services of Sangon Biotech (Shanghai, China).

### Phylogenetic analysis

A rooted tree was generated using MEGA 5.0, with the maximum likelihood (ML) method according to the Jones–Taylor–Thornton (JTT) model^30^. The alignments of protein sequences were generated by Clustal X.2^31^. VGSC of *Homo sapiens* (NP_066287.2) and *Mus musculus* (NP_001092768.1) were used as outgroups. The variable regions between the H1 and H2 subunits of the three aphid species—*R. padi*, *A. pisum*, and *M. persicae*—were excluded, and the two subunits were combined for better alignment. Bootstrapping of the ML analysis was conducted using 1000 replicates.

### *Rpvgsc* expression in *R. padi* according to developmental stage

For analysis of *Rpvgsc* expression at different developmental stages, individuals (5 mg) of each stage from RpSS strain were used to isolate total RNA as described above. Three independent extracts were generated. The first-strand cDNA of each RNA sample for qPCR was synthesized using 1.5 μg of total RNA using an M-MLV reverse transcriptase cDNA Synthesis Kit (Promega, Madison, WI, USA) in 20 μL reaction mixtures as described by the manufacturer. Primers for RT-qPCR were listed in Table 1. β*-actin* was used as the house-keeping gene in the analysis^32^,^33^. The specificity of primer pairs (QH1 and QH2 for target gene amplification and *β-actin* as the endogenous control) was tested and confirmed by sequecing basing on preliminary experiment (data not shown). qPCR reactions were performed in a 20 μL total reaction volume including 10 μL of 2 × SYBR 1 Premix^®^ EX Taq^TM^ (Takara) master mix, 0.8 μL each of gene-specific primers (Table 1), 2 μL of cDNA template, and 6.4 μL of ddH_2_O. Samples without reverse transcriptase enzyme or cDNA template were used as negative controls. Three technical replicates were run for each sample. Reactions were carried out using an iQ5^TM^ (Bio-Rad, Hercules, CA, USA) according to the operation manual. The PCR parameters were as follows: 3 min at 95 °C; 40 cycles of 10 s at 95 °C, of 20 s at 58 °C, and of 20 s at 72 °C; followed by 15 s at 55 °C (gradient rising to 95 °C at 0.5 °C intervals) to generate a melt curve. At least three individual samples were prepared, and each sample was analyzed in triplicate.

### *Rpvgsc* expression after induction with beta-cypermethrin

According to assays of the toxicity of beta-cypermethrin (96%, Yancheng Nongbo Biotechnology Co., Ltd., Jiangsu China) to *R. padi* (data not shown), we used sublethal LC_10_ (0.3987 mg/L) and LC_30_ (0.9280 mg/L) concentrations of beta-cypermethrin and assessed the response of *Rpvgsc* genes. Beta-cypermethrin was dissolved in acetone as a stock solution (10 g/L). Then, the stock solution was further diluted to the LC_10_, LC_30_ and LC_50_ beta-cypermethrin solution using 0.1% Triton X-100 solution, with 0.0040% (v/v) acetone in LC_10_ solution, 0.0093% (v/v) acetone in LC_30_ solution, and 0. 0167% (v/v) acetone in LC_50_ solution, respectively. The leaf-dipping method was adopted for the reagent^15^,^33^. Wheat leaves with apterous adult aphids were dipped in the insecticide solutions for 10 s. The leaves were then removed from the solution, and residual droplets on the leaf were adsorbed using clean, dry filter paper. Then, the leaves were transferred to plastic Petri dishes lined with moistened filter paper. Each insecticide concentration was assayed in triplicate, each using 100 apterous adult aphids. Water was used as a control. Live aphids treated with insecticide or water were collected at 12, 24, and 36 h for determination of mRNA expression levels. qPCR was performed to assess insecticide-induced *Rpvgsc* expression. The qPCR method was identical to that described above.

### Double-stranded RNA synthesis of H1 and H2 subunits

Sequence-specific primers of H1 and H2 subunits (Table 1) were synthesized to amplify target sequences. The PCR fragments was gel-purified (Promega, Madison, WI), cloned into the pGEM-Teasy Vector (Promega, Madison, WI) and transformed into *Escherichia coli* JM109. The recombinant plasmids were sequenced by Sangon Biotech (Shanghai, China) to ensure the reliability of these primers. To synthesize H1-dsRNA, two separate PCR reactions were performed using a sequence-specific primer and a primer by attaching T7 promoter sequences at 5′ terminal (Table 1). The same method was used to synthesize H2-dsRNA. Then, the PCR products were purified and utilized to synthesize dsRNA using the T7 RiboMAX™ Express RNAi System (Promega, Madison, WI, USA) according to the manufacturer’s instructions. The dsRNAs were dissolved in nuclease-free water. The purity and concentration of dsRNAs were examined the biophotometer (Eppendorf BioPhotometer Plus, Eppendorf, Germany). The dsRNAs were stored at −80 °C until use. The dsGFP was synthesized and used as a control.

### RNAi of H1 and H2 subunit genes and insecticide bioassays

A volume of 41.4 nL dsRNA (4.1 μg/μL) was injected into body cavity of each one-day-old apterous adult aphid by a micro glass needle using automatic nanoliter injector (Märzhäuser, Wetzlar, Germany). Injection location on the aphids were at the suture joining the ventral mesothorax and metathorax^34^. The mechanical injuries caused about 20% mortality to the aphids. After injection, each aphid alive was reared on a wheat leaf in a plastic petri dish (9 cm in diameter) under condition of 24 °C, 70% relative humidity and a 16:8 h L: D photoperiod.

To determine the reduction of transcription levels of H1 and H2 subunit genes and to determine the time of highest interference efficiency, ten survival adults were randomly collected at 24 h, 48 h, 72 h, 96 h or 120 h after injection. qPCR was used to analyze the transcription levels of H1 and H2 subunit genes of the survival aphid collected. The qPCR primers were from a separate region of H1 and H2 subunit genes to those used for RNAi. Three replications were carried out, with at least 200 aphids per treatment.

After the time of the highest RNAi efficiency for each of the two genes were determined, about 200 one-day-old apterous adult aphids were randomly collected and divided into two groups, and H1-dsRNA and dsGFP were injected into the aphids of two groups, respectively. Then, the LC_50_ (1.667 mg/L) concentrations of beta-cypermethrin was applied on each survival aphid from the two groups at time of highest interference efficiency using the aforementioned leaf-dipping method, and around 80 naive aphids were used as control. The mortality of the aphids in the three treatments was assessed 24 h after exposure to the chemical. Three replicates were carried out. The same method was used to examine the effect of H2 subunit gene RNAi on the sensitivity of *R. padi* to beta-cypermethrin.

### Data analysis

DNA sequences were analyzed using ClustalX (http://www.clustal.org/). A BLAST search was performed at the NCBI Website http://blast.ncbi.nlm.nih.gov/Blast.cgi. qPCR results were analyzed using the2^−ΔΔCt^ method^35^. Bioassay data were analyzed using using SPSS 16.0 software (SPSS Inc., Chicago, IL, USA). A two tailed *t* test at the significance level 0.05 was used to test whether the expression level of *Rpvgsc1* and *Rpvgsc2* was significant different between aphids with and without exposure to beta-cypermethrin. Mortality rate data (percentage) were transformed using arcsine square-root transformation, and then the transformed data were subjected to ANOVA. Data of relative expression was also subjected to ANOVA. All ANOVA was followed by Tukey’s honest significant difference (HSD) multiple comparisonst using ProStat software (Poly Software International, Pearl River, NY).

## Additional Information

**How to cite this article**: Zuo, Y. *et al*. Expression patterns, mutation detection and RNA interference of *Rhopalosiphum padi* voltage-gated sodium channel genes. *Sci. Rep.* **6**, 30166; doi: 10.1038/srep30166 (2016).

## Figures and Tables

**Table 1:** Primers used for RT-PCR, quantitative PCR and RNA interference of *Rpvgsc*.

Fragment	Forward (5′ to 3′)	Reverse (5′ to 3′)
H1-1	CGTCTGTATCGTGTGGTGTCGG	TAGTCGGAGTTGGGGGCATTAT
H1-2	TGCTGACACCGATTCTT	ATCCCAGTAATGCGAAC
H1-3	AACTCCGACTATTGAAGCGT	CAAGATAAAACGAGCCGAG
H1-4	GTTCGCATTACTGGGATT	CCTTGGTCATAGGTGGTTT
H1-5	TATGCTGACGACTCAAACGC	CGAAGCAAACGAAATGAACG
H1-6	GAAGCCGCTCTGAAACTG	TGACGCCTGGACCGATA
H1-7	TGCCTACGGCTGACAGTGAC	TCACATCTGGCACGGTAGG
Full H1	GCCGACGAGGGTATAAGAGGT	TAAACAGCGTGGTGTGTAGCC
H2-1	CCGCCAATCAGAATAGTAATGT	AATGGCGTTTCATCGTCAC
H2-2	TACATTGCGGTCATCCTG	GTTTTAGACGTCGGCGAG
H2-3	GGCTGGTGACGATGAAAC	CAAACCCGCTAAGGAAAG
H2-4	TAAAGAGCCGTGGAACCT	CGGTCTCAATGATGGGAT
Full H2	GGAACTCCTGGATGCTGGAATCA	CGAGTGAATGACAACCTTGTGACC
QH1	CCATTGCTGACACCGATTCTT	CATCTTCGTCCTCGTCCTCAT
QH2	GGAACCGCCATTGAAGATCCAC	TCACGCATACGCCACAGAGAT
β-actin	GCCCAATCCAAAAGAGGTAT	TCAAAGGTGCTTCCGTTAGT
RpNa	CCGTAGTAGAACTGCTCTCCGC	TGTC AGCCGTAGGCACCGAT
ds-H1	CGGTTCGAGGCACAGCAGTTAC	ACTTGAGCCACGGCCATCCA
T7-H1	TAATACGACTCACTATAGG CGGTTCGAGGCACAGCAGTTAC	TAATACGACTCACTATAGG ACTTGAGCCACGGCCATCCA
ds-H2	AACGCTCCTTCGAGTGGTCAGA	ACTGCCCGTGATACCCATCTCT
T7-H2	TAATACGACTCACTATAGG AACGCTCCTTCGAGTGGTCAGA	TAATACGACTCACTATAGG ACTGCCCGTGATACCCATCTCT
dsGFP	GTGTTCAATGCTTTTCCCGT	CAATGTTGTGGCGAATTTTG
T7GFP	TAATACGACTCACTATAGG GTGTTCAATGCTTTTCCCGT	TAATACGACTCACTATAGG CAATGTTGTGGCGAATTTTG

**Table 2:** The seven field populations of *R*. *padi* sampled in China.

Province	Population	Code	Latitude, Longitude	Date
JiLin	Baicheng	JLB	45°39′N, 122°52′E	July, 2013
GanSu	LanZhou	GSL	36°05′N, 103°41′E	June, 2013
HeNan	NanYang	HNN	33°14′N, 112°36′E	April, 2013
HeBei	BaoDing	HEB	38°49′N, 115°26′E	June, 2013
ShanXi	TaiGu	SXT	37°25′N, 112°34′E	May, 2013
ShanDong	TaiAn	SDT	36°06′N, 117°14′E	May, 2013
AnHui	ChuZhou	AHC	32°21′N, 118°20′E	April, 2013
